# The epidemiology of headaches among patients with epilepsy: a systematic review and meta-analysis

**DOI:** 10.1186/s10194-020-1074-0

**Published:** 2020-01-10

**Authors:** Bereket Duko, Mohammed Ayalew, Alemayehu Toma

**Affiliations:** College of Medicine and Health Sciences, Hawassa University, Hawassa, Ethiopia

**Keywords:** Prevalence, Headaches, Epilepsy, Seizure, Systematic review, Meta-analysis

## Abstract

**Background:**

Headache is the symptom of pain in the face, head or neck that causes disability in most people with medical and neurological disorders. It frequently co-occurs with most chronic diseases such as epilepsy and significantly impacts the quality of life. However, epidemiologic data from different studies showed different rates of prevalence. Therefore, we conducted this review to summarize the available epidemiologic evidence on the topic and formulate recommendations for future research and clinical practice.

**Methods:**

We followed the preferred reporting items for systematic review and meta-analysis (PRISMA) guidelines. We systematically searched the literature using popular databases such as PubMed, EMBASE, Psych-INFO, and SCOPUS. We further scanned the reference lists of the eligible studies to supplement our electronic search. The Comprehensive Meta-Analysis software version 3.0 (CMA 3.0) was used to conduct a meta-analysis. Subgroup and sensitivity analysis were performed and Cochran’s Q- and the I^2^- test were used to assess the source of heterogeneity. The funnel plot and Egger’s regression tests were used to assess potential publication bias.

**Results:**

A total of 17 studies conducted both in developed and developing countries including 5564 study participants were combined in this meta-analysis. The pooled estimated prevalence of headache among patients with epilepsy was 48.4%. The pooled estimated prevalence of Inter-Ictal headache (IIH) (42.2%) and Postictal headache (PIH) (43.1%) were higher when compared to tension-type headache (TTH) (26.2%), migraine with aura (26.0%) and migraine without aura (10.4%). The pooled prevalence of headache was 50.6% and 49.5% for developed and developing countries respectively. The pooled prevalence of headache among patients with epilepsy was considerably higher among females (63.0%) when compared to males (33.3%). Moreover, the pooled estimated prevalence of headache among patients with epilepsy was ranging from 46.0% to 52.2% in a leave-one-out sensitivity analysis.

**Conclusion:**

The pooled estimated prevalence of headache among patients with epilepsy was considerably high (48.4%). Screening and appropriate management of headaches among patients with epilepsy are warranted.

## Background

Neurological disorders are emerging challenges to health care systems and requiring further study, government as well as social engagement, and improvements in health care infrastructure [1]. Epilepsy is one of the neurologic disorders which is characterized by repeated seizure attacks which result from paroxysmal uncontrolled discharges of neurons [2–5]. The World Health Organization defines epilepsy as having two or more unprovoked seizures [2].

Epilepsy affected around 50 million people worldwide in 2019 [2]. Of these, 80% of people with epilepsy live in low- and middle-income countries [2, 6, 7]. A systematic review and meta-analysis conducted in 2017 reported that the point prevalence of active epilepsy was 6.38 per 1000 persons while the lifetime prevalence was 7.60 per 1000 persons [8]. Further, the annual cumulative incidence of epilepsy was 67.77 per 100,000 persons while the incidence rate was 61.44 per 100,000 person-years [8]. Finding from another study also revealed that the lifetime incidence of epilepsy is ranging from 1 to 26 with a peak age ranges from 30 to 50 years [9].

Headache is the symptom of pain in the face, head, or neck that causes disability in most people with medical and neurological disorders [10]. There are different types of headaches that can occur in individuals but generally can be classified as primary and secondary headaches [11]. Primary headaches broadly include migraines and tension-type headaches [12]. Migraines are characterized by pulsing head pain, nausea, and sensitivity to light and sound whereas tension-type headaches present with non-pulsing “band-like” pressure on both sides of the head but not accompanied by other symptoms [13].

Headache frequently co-morbid with most chronic diseases such as epilepsy [14–17]. For instance, a review conducted to see the relationship between headache and epilepsy reported the comorbidity of headache and epilepsy as a result of common genetic mutations and clinical features [18, 19], but the suggested link is not revealed conclusive evidence of a real causal association [20]. Further, studies also suggested that there are genetic relationships [19] as well as common underlying pathophysiological mechanisms including the imbalance between excitatory and inhibitory neurotransmitters in epilepsy and headache, especially for migraine [18, 21]. Proposed theories for shared etiologies include ion channel dysfunction, glutamatergic mechanisms, and mitochondrial dysfunction [22, 23]. These suggest that the cause of headache and epilepsy are multifactorial and hence need different diagnostic and interventional approaches [24].

Epidemiologic evidence from different studies reported different rates of prevalence of headache among epileptic patients [25, 26]. For example, a cross-sectional study conducted in China to assess the prevalence of headache among patients with epilepsy reported 60.1% [25]. In contrast, a study assessed the prevalence of headache among epileptic patients in Japan revealed 23% [26]. Thus, estimating the burden of headaches among patients with epilepsy is critically important. Even though a systematic review and meta-analysis has been conducted in 2017 on a similar area of study [27], however, it only assessed the comorbid relationship between migraine and epilepsy [21]. In spite of that, the current review tried to investigate the pooled prevalence of headaches such as migraine, tension-type headaches, and others among patients with epilepsy to summarize the available epidemiologic evidence on the topic and formulate recommendations for future research as well as clinical practice.

## Methods

### Study design and search process

This systematic review and meta-analysis followed the preferred reporting items for systematic review and meta-analysis (PRISMA) guidelines [28]. We reviewed research articles that assessed the prevalence of headaches among patients with epilepsy. We systematically searched the literature using common databases such as PubMed, EMBASE, Psych-INFO, and SCOPUS. Medical Subject Headings (MESH) terms were used to perform the electronic database search in PubMed: *“(headache OR migraine OR tension-type headache OR Headache on seizure-day OR postictal headache OR ictal headache OR primary headaches OR cluster headaches) AND (prevalence OR magnitude OR epidemiology OR incidence)) AND (Epilepsy OR Seizure disorder OR convulsion OR seizure attack OR paroxysm)”.* The EMBASE, Psych-INFO, and SCOPUS databases were also comprehensively searched by applying the search terms used in PubMed for each database accordingly. Further, we also boosted our literature search through a manual search of the reference lists of eligible articles.

### Eligibility criteria

Studies were included in the review if they fulfill the following inclusion criteria: observational studies including cross-sectional design; conducted among patients with epilepsy or seizure disorders, studies that were published in the English language and determined the prevalence of primary headaches among patients with epilepsy. However, we excluded commentaries, letters, duplicate studies, editorials, reviews, and short communications as they did not satisfy the eligibility criteria.

### Methods for data extraction and quality assessment

The data extraction from the relevant studies was employed by two independent reviewers. We extracted the following information from each study: the name of the first author, the year of publication, study setting, and design, sample size, prevalence, and tools used to estimate the magnitude of AUD, type of headaches reported and the reported magnitude by gender of the participants. Disagreements raised during data extraction were solved by discussion. The Newcastle-Ottawa Scale (NOS) was used to check the quality of the included studies in the meta-analysis [29, 30]. The data measurement tools used to assess types of headaches, sample size, methodological quality, types of headache, sample representativeness and comparability between participants were the domains of the NOS scale to assess the quality of the included studies.

### Definition of terms

Headache is the symptom of pain in the face, head, or neck that causes disability in most people with medical and neurological disorders [10]. In this review, headache was considered when the studies investigated and reported the prevalence of primary headaches such as migraine (with or without aura), tension-type headache, Ictal headache, and post-ictal headache among patients with epilepsy based on any standardized and validated screening instruments used to assess headaches such as Migraine Screening –Questionnaire (MSQ), the International Classification of Headache Disorders 2nd or 3rd edition (ICHD-2, 3), the Headache-Attributed Lost Time (HALT) and the six-item Headache Impact Test.

### Data synthesis and analysis

A meta-analysis was conducted by a Comprehensive Meta-Analysis software version 3.0 (CMA-3.0). The random-effect model for meta-analysis was employed to pool the overall prevalence of primary headaches among patients with epilepsy. The magnitude of statistical heterogeneity between the eligible articles was checked by Q-statistic and the I^2^-statistics [31] and values of 25, 50 and 75% were used to represent low, medium and high quality respectively [32]. Further, we also conducted a subgroup and sensitivity analysis to check potential source bias among the included studies. The types of primary headaches assessed, the country study conducted, and study design and the gender of study participants (male or female) were used as a moderator to assess subgroup and sensitivity analysis. The funnel plot and Egger’s regression tests were used to assess potential publication bias [33].

## Results

### Identification of studies

**Fig. 1:**
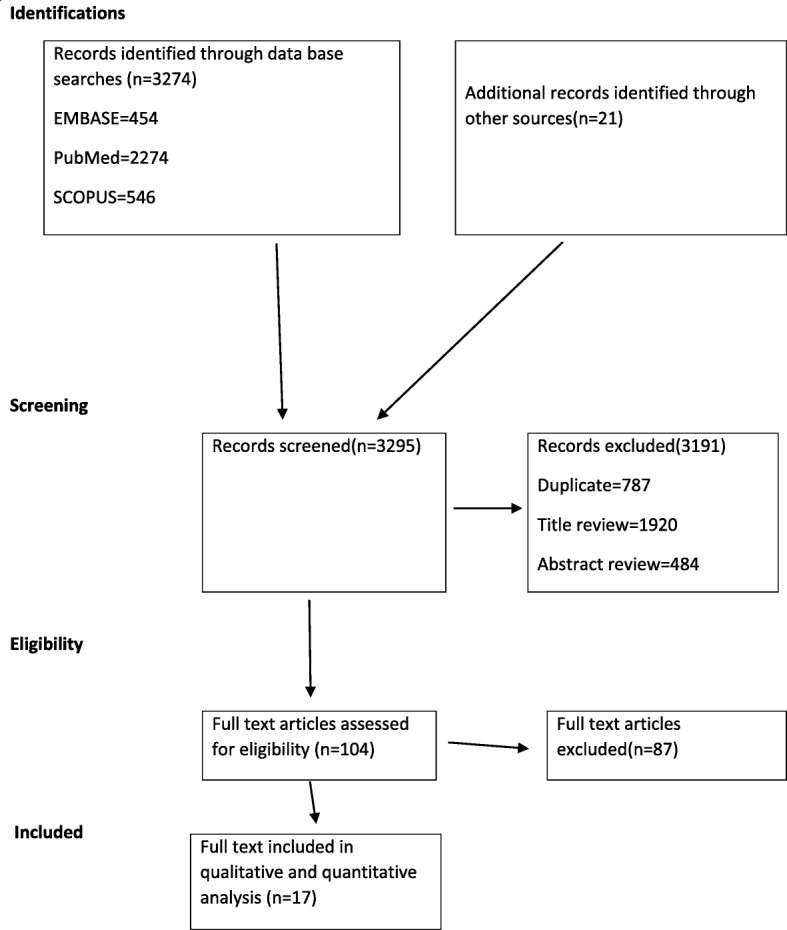
PRISMA flowchart of review search

The electronic database along with additional manual reference searches resulted in a total of 3295 research articles. Of these, 104 articles were retrieved for further screening and 87 articles were excluded, 17 research articles were included in the final meta-analysis (see Fig. 1).

### Characteristics of included studies

A total of seventeen studies conducted both in developed and developing countries including 5564 study participants were included in this meta-analysis. The studies included in this systematic review were published between 2004 and 2019 and the sample size ranging between 86 in Turkey and 1109 in China. Of 17 studies included the final meta-analysis, one from the USA [34], one from India [35], three from Iran [36–38], one from Japan [26], one from Taiwan [35], one from Italy [36], three from China [25, 39, 40], one from Turkey [41], one from Lithuania [42], one from Montenegro [43], one from Bangladesh [44] and two from Egypt [45, 46]. Regarding types of headaches assessed among patients with epilepsy, nine studies assessed tension-type headache, fifteen studies assessed migraine with aura, three studies assessed migraine without aura, and seven studies assessed both Ictal headache (IH) and Postictal headache (PIH). The studies included in the review used Migraine Screening –Questionnaire (MSQ), the International Classification of Headache Disorders 2nd and 3rd edition (ICHD-2, 3), the Headache-Attributed Lost Time (HALT) and the six-item Headache Impact Test (HIT-6) to assess primary headaches among patients with epilepsy (See Table 1).

**Table 1:** Characteristics of the included studies on prevalence of headache among patients with epilepsy

First author, date	Country	Sample size	Study design	Data collection tool	Prevalence (%)		Prevalence of any headache (%)	Prevalence (%)				
					Male	Female		MA	MOA	TTH	IIH	PIH
Erin K, 2017 [34]	USA	160	Cross-sectional	Self-report	NA	NA	40.00	NA				
Singla et al., 2019 [35]	India	123	Cohort	Self-report	NA	NA	47.10	21.10		26.00		
Sayena J et al., 2015 [36]	Iran	150	Cross-sectional	MSQ	NA	NA	32.60	15.30	17.30			
Ashjazadeh N, 2015 [37]	Iran	100	Cross-sectional	ICHD-II	48	52	54.00	15.00		39.00	42.53	31.48
Fattahzadeh AG et al., 2017 [38]	Iran	900	Cross-sectional	HIS	NA	NA	85.20	27.90		48.20		
Ito M et al., 2004 [26]	Japan	364	Cross-sectional	ICHD-III	NA	NA	40.40	26.00			50.00	40.40
Inn-Chi L., 2018 [47]	Taiwan	476	Cross-sectional	ICHD-III	NA	NA	9.70	9.70				
Mainieri et al., 2015 [39]	Italy	388	Cross-sectional	ICHD-II	NA	NA	53.90	26.30		19.10	48.50	19.10
Zhang et al., 2018 [37]	China	339	Cross-sectional	ICHD-III	17	29	23.00	23.00				
Gökhan Ö et al., 2010 [41]	Turkey	86	Cohort	Self-report	NA	NA	47.60	13.90	12.80	16.30		
Mameniškiene R et al., 2016 [42]	Lithuania	289	Cross-sectional	HALT	42	62	83.20	31.70		39.00	77.90	
Wang et al., 2014 [25]	China	1109	Cross-sectional	ICHD-II	57	64	60.10	60.20		30.30	11.70	34.10
Slavica V. et al., 2012 [43]	Montenegro	259	Cross-sectional	ICDH II	20	85	48.00	15.44				28.00
Shamim R..,2018 [44]	Bangladesh	376	Cross-sectional	NA	NA	NA	80.60	NA				80.60
Kwan P et al., 2008 [38]	China	227	Cohort	HIT-6	68	NA	22.00	4.80	3.10	7.00	19.80	
Yosria AHA. et al., 2017 [41]	Egypt	118	Case control	ICHD-III	31	69	24.60	79.30				
Sayed MA et al*, 2019* [42]	Egypt	100	Cross-sectional	ICHD-III	27	73	78.00	70.60		25.58	50.00	66.50

Foot notes: *MSQ* Migraine Screening Questionnaire, *ICHD-II* The International Classification of Headache Disorders 2nd edition, *ICHD-3* The International Classification of Headache Disorders 3rd edition, *HALT* The Headache-Attributed Lost Time, *HIT-6* The six-item Headache Impact Test, *IHS* International Headache Society, *MA* migraine with aura, *MOA* Migraine without aura, *TTH* Tension type headache, *PIH* Post ictal headache, *IIH* Inter Ictal headache

### Quality of included studies

The Newcastle Ottawa Scale (NOS) was used to check the quality of the included studies. Of 17 studies included in the review, 14 studies were of high quality (NOS score > 8), 2 studies were moderate quality (NOS score between 6 and 7) and 1 study was low-quality studies (NOS score < 5) (See Additional file 1).

### The prevalence of headaches among patients living with epilepsy (meta-analysis)

**Fig. 2:**
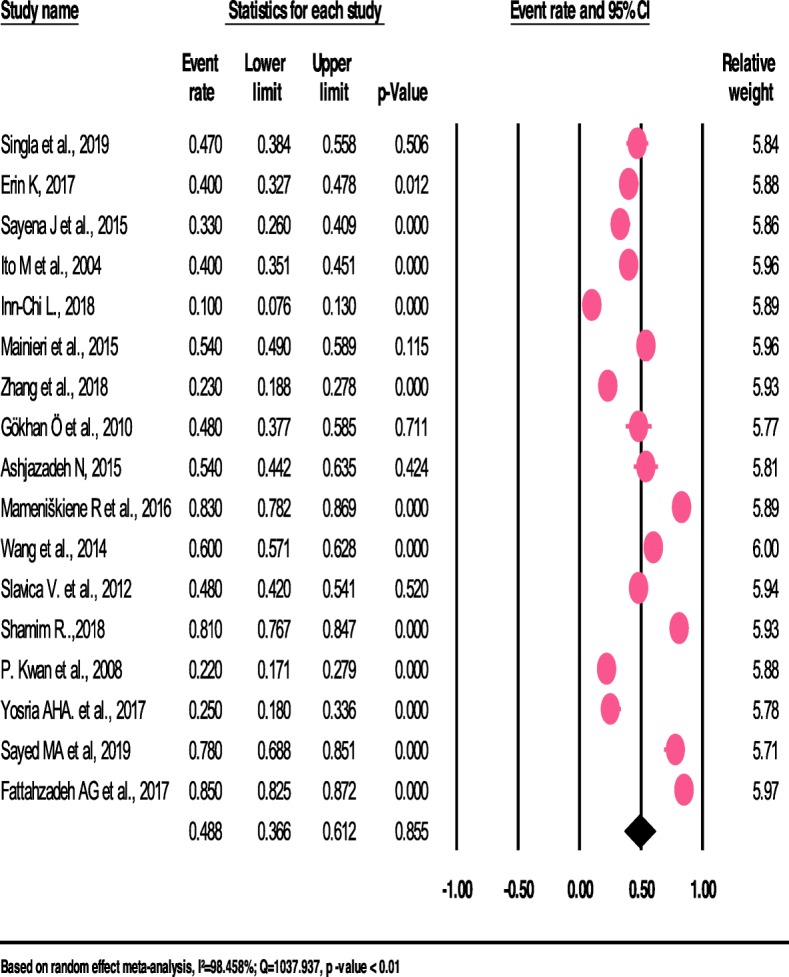
The prevalence of headache among patients with Epilepsy: a meta-analysis (forest plot)

The pooled prevalence estimate of headache among patients with epilepsy was 48.4% (95% CI; 36.6–61.2). We found an apparent heterogeneity among included studies in this meta-analysis (*I*^*2*^ = 98.458%; *p* <  0.001) (See Fig. 2).

### Subgroup and sensitive analysis

The available epidemiologic evidence was diverse by the types of headaches present in patients with epilepsy, the country in which a study conducted, the methodological design and the gender of study participants (male or female).

**Fig. 3:**
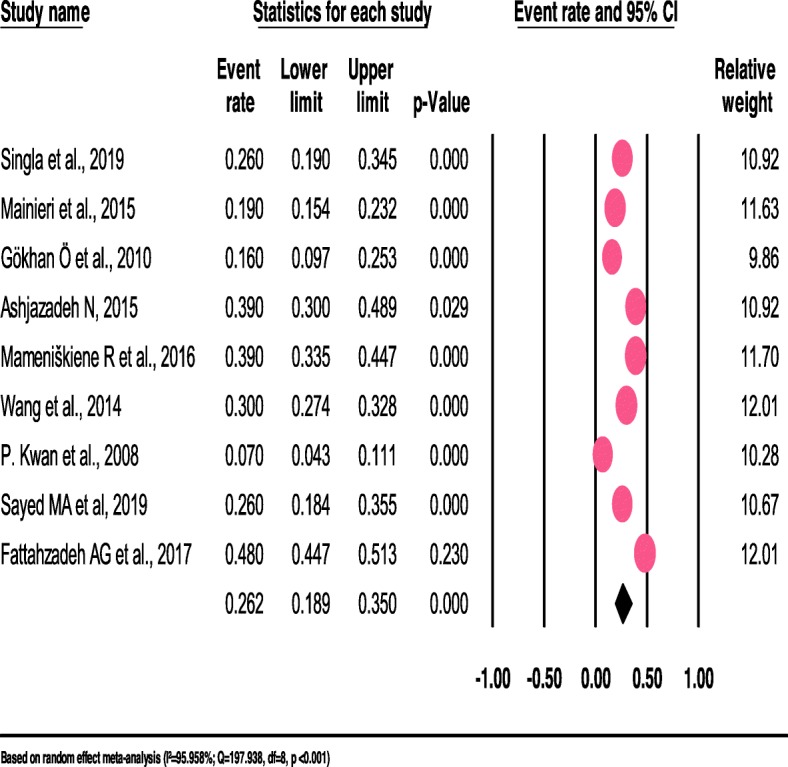
The prevalence of tension-type-headache among patients with epilepsy: a meta-analylsis (forest Plot)

**Fig. 4:**
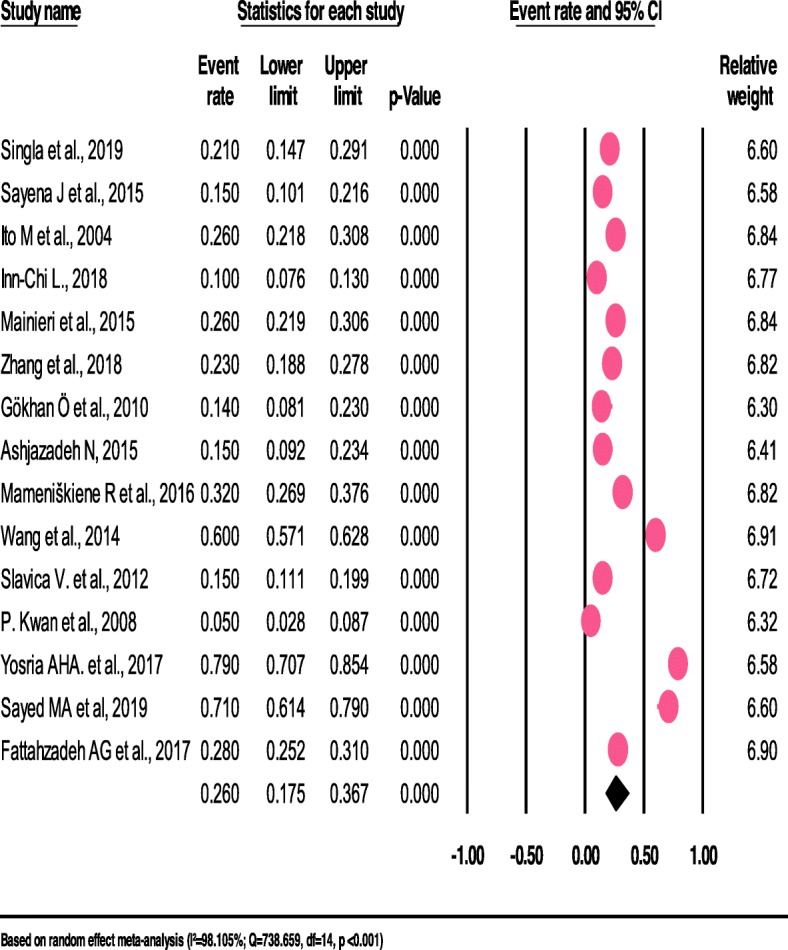
The prevalence of migraine-with-aura among patients with epilepsy: a meta-analysis (forest plot)

The pooled prevalence of headaches among patients with epilepsy differed when types of headaches differ. Thus, the pooled prevalence of Inter Ictal headache (IIH) (42.2%) and Postictal headache (PIH) (43.1%) among patients with epilepsy were higher when compared to tension-type headache (TTH) (26.2%) (See Fig. 3), migraine with aura (26.0%) (See Fig. 4) and migraine without aura (10.4%).

The pooled prevalence of primary headaches among patients living with epilepsy did not differ in the subgroup analysis of developing and developed countries as a moderator. For example, the pooled prevalence of headaches was 50.6% (95%CI 35.9–65.3) and 49.5% (95%CI 38.1–60.9) for developed and developing countries respectively. The variation between the studies was statistically significant (*P* <  0.001).

We also performed a subgroup analysis using the methodological design studies conducted as a moderator. The prevalence of primary headaches was higher in the studies that used a cross-sectional study design (53.4%) when compared to the studies that used cohort (37.9%) and case-control study (25.0%) designs.

We further conducted a subgroup analysis using the gender of the study participants as a moderator. The pooled prevalence of headaches among patients with epilepsy was considerably higher among females (63.0%) when compared to males (33.3%). The heterogeneity was significant for both studies that assessed the prevalence of headaches in females (*I*^2^ = 96.958%, *p* <  0.001) and (*I*^*2*^ = 97.451%, *p* <  0.001) (See Table 2).

**Table 2:** Subgroup and sensitivity analysis for the prevalence of headaches among patients with epilepsy

Subgroups	No. of studies	Prevalence (%)	95% CI	Heterogeneity within the study (I^2^, Q and *p*-value)		
				Q-value	I^2^ (%)	*P*-value
Types of Headache						
Tension type headache (TTH)	9	26.2	18.9–35.0	197.938	95.958	< 0.01
Migraine with aura	15	26.0	17.5–36.7	738.659	98.105	
Migraine without aura	3	10.4	5.20–19.7	11.064	81.924	
Ictal headache (IH)	7	42.2	26.8–59.2	523.957	98.664	
Post ictal headache (PIH)	7	43.1	30.9–56.3	348.352	97.991	
Countries						
Developed	6	50.6	35.9–65.3	142.400	96.489	< 0.01
Developing	11	49.5	38.1–60.9	885.368	98.871	
Study design						
Cross-sectional	13	53.4	39.2–67.0	909.646	98.681	< 0.01
Cohort	3	37.9	21.3–57.8	30.119	93.360	
Case-control	1	25.0	18.0–33.6	0.000	0.000	
Gender						
Male	7	33.3	21.1–48.3	235.349	97.451	< 0.01
Female	7	63.0	49.1–75.1	197.232	96.958	

Moreover, we conducted a leave-one-out sensitivity analysis to further explore the source heterogeneity. The pooled prevalence of headaches among patients with epilepsy was ranging from 46.0% (35.0–57.4) to 52.2% (40.8–63.4) in the leave-one-out sensitivity analysis (See Table 3).

**Table 3:** Leave-one-out-sensitivity analysis of prevalence of headache among patients: prevalence and 95% confidence

Study excluded	Prevalence (%)	95%CI
Singla et al., 2019	48.9	36.2–61.8
Erin K, 2017	49.4	36.6–62.3
Sayena J et al., 2015	49.9	37.2–62.6
Ito M et al., 2004	49.4	36.4–62.5
Inn-Chi L., 2018	52.2	40.8–63.4
Mainieri et al., 2015	48.5	35.3–61.9
Zhang et al., 2018	50.7	38.3–62.9
Gökhan Ö et al., 2010	48.9	36.2–61.7
Ashjazadeh N, 2015	48.5	35.8–61.4
Mameniškiene R et al., 2016	46.3	34.2–58.9
Wang et al., 2014	48.1	34.2–62.4
Slavica V. et al., 2012	48.9	35.9–62.0
Shamim R..,2018	46.5	34–3-59.1
P. Kwan et al., 2008	50.7	38.3–63.1
Yosria AHA. et al., 2017	50.4	37.9–63.0
Sayed MA et al., 2019	46.9	34.5–59.6
Fattahzadeh AG et al., 2017	46.0	35.0–57.4

### Publication bias

**Fig. 5:**
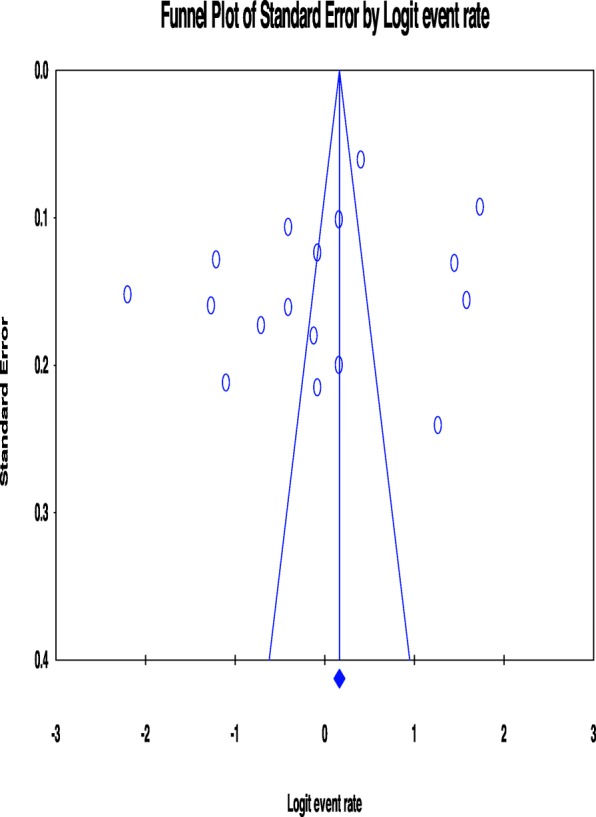
Funnel plot showing no publication bias among studies included in the review

For the overall meta-analysis of the prevalence of headache among patients with epilepsy, both funnel plot and Egger’s regression tests revealed no evidence of potential publication bias (B = -6.59, SE = 5.23, *P* = 0.23) (See Fig. 5).

## Discussion

To our knowledge, this is the first systematic review and meta-analysis that assessed the prevalence of headaches among patients with epilepsy globally. In this review, the pooled prevalence of headaches among patients with epilepsy was 48.4%. The available epidemiologic evidence was diverse by the types of headaches present in patients with epilepsy, the country in which a study conducted, the methodological design and the gender of study participants (male or female). Further, the majority of included studies were of high methodological quality.

The pooled prevalence estimate of headache among patients with epilepsy in the current systematic review and meta-analysis (48.4%) was in line with the World Health Organization (WHO) report of 2016 which estimated the prevalence of headache disorders among adults was 50% [48]. However, the pooled prevalence of headache among patients with epilepsy was higher than the reported prevalence of headache in the general population. For example, the studies conducted to assess the prevalence of primary headache disorders in a geriatric population (age > 60 years) in a rural area of Northern China reported 10.3% [49]. This study finding was supported by a community-based cross-sectional study conducted to assess the burden of primary headache disorders among Kuwaiti children and adolescents and revealed 19.4% [50]. The finding from a study conducted to assess the prevalence of headache among medical students in Saudi Arabia also reported of 41.66% [51]. Further, a study that described the clinical characteristics of primary headaches occurring in a group of HIV-infected individuals and reported 38% [52]. The discrepancy in the prevalence of headache may be due to the difference in the study population, setting and, also primary headaches such as migraines share some common signs and symptoms with epilepsy, therefore, this may increase the prevalence of headaches among patients with epilepsy. In addition, there are numerous ways in which common genetic factors such as SCN1A mutations that predispose persons to the development of headaches and epilepsy [27, 53] and the corresponding mutations may result in higher prevalence of headaches among patients with epilepsy [54]. A study conducted to assess the contribution of a shared genetic susceptibility to migraine and epilepsy reported that the prevalence of a history of migraine was significantly increased in participants with affected first degree relatives [55]. Further, shared environmental factors e.g. head injury, may result in brain hyper-excitability [56], and these may play a great role in the variation of prevalence of headache among patients with epilepsy when compared to the general population.

In this review, the pooled prevalence of migraine and tension-type headache in patients with epilepsy was 26.0% and 26.2% respectively. The pooled estimated prevalence of migraine and tension-type headaches were higher than pre-Ictal headaches reported by different studies [25, 39]. Further, the pooled prevalence of migraine was higher than the prevalence of migraine and tension-type headache in the general population. For example, a research article published simultaneously by Cephalalgia Headache and the Journal of Headache and Pain in 2013 showed that the global prevalence of migraine in the general population was 14.7% [57, 58]. Also, a systematic review conducted in 2010 to assess the prevalence of chronic migraines reported 0–5.1% in the general population [59]. This may be due to the comorbidity of migraine and epilepsy in which the two disorders experience common symptoms, risk factors, and drug therapy [25]. Findings from previous studies support the hypothesis of cortical excitability as a plausible mechanism underlying their pathology [60]. Further, there is corroboration from the scientific study of the nervous system that cortical spreading depression and an epileptic focus may exacerbate each other [61].

In our subgroup analysis, the pooled prevalence of headaches among patients with epilepsy was considerably higher among females (63.0%) when compared to males (33.3%). Data from different epidemiologic studies reported that female manifests high prevalence of migraine [62]. The same study also showed that female experiences more frequent, longer-lasting and more intense attacks than male [61, 62]. Further, scientific evidence has shown that primary headaches such as migraines are associated with sex hormones [61]. Finding from this study showed that sex hormones in females affect cells around the trigeminal nerve as well as connected blood vessels in the head. The estrogens, which are hormones that are responsible for reproductive and sexual development in females, at their highest levels in the females of childbearing age are particularly important for sensitizing these cells to migraine triggers [63]. Furthermore, a study included a total of 2082 study participants to investigate the effect of gender on the headache manifestations in migraine patients and reported that the headache intensity in males changed in an age-dependent manner and these variations were not seen in males [64].

The pooled prevalence of primary headaches among patients living with epilepsy did not differ in the subgroup analysis of developing and developed countries. For example, the pooled prevalence of primary headaches was 50.6% and 49.5% for developed and developing countries respectively. These may indicate the risk factors of headache among patients with epilepsy are common irrespective of the countries’ socio-economic status and suggesting some common biological factors are explaining the co-occurrence.

## Strength and limitations

This systematic review and meta-analysis has the following strengths; we used a predefined search strategy and two independent reviewers conducted data extraction and quality appraisal to minimize reviewer bias, conducted sensitivity and subgroup analysis using types of headaches, the countries the study based and the gender of the participants as a moderator. Nevertheless, this systematic review has the following limitations. We got a small number of articles in the subgroup analysis which could decrease the accuracy of the estimate. We did not analyze the data on children due to lack of sufficient literature. Furthermore, some studies included in the review did not use standardized and validated data measurement tools. These may overestimate the prevalence of headache in the studies that used a non-standardized data collection tool. Moreover, the review included studies published in the English language only. This may under- or over-estimate the pooled prevalence of headache among patients with epilepsy.

## Conclusion

The pooled prevalence estimates of headache among patients with epilepsy was considerably high when compared to the general population. The pooled prevalence of Inter Ictal headache (IIH) and Postictal headache (PIH) were higher when compared to tension-type headache (TTH), migraine with aura and migraine without aura. The pooled prevalence of headache was similar for developed and developing countries. The pooled prevalence of headache among patients with epilepsy was considerably higher among females when compared to males. Screening and appropriate management of headaches among patients with epilepsy are warranted.

## Supplementary information

**Additional file 1.** The quality of studies included in systematic review and meta-analysis.

## Data Availability

All data generated or analyzed data in study are included in this article.

## Abbreviations

HALT: The Headache-Attributed Lost Time
HIT-6: The six-item Headache Impact Test
ICHD-III: The International Classification of Headache Disorders 3rd edition
IHS: International Headache Society
IIH: Inter Ictal headache
MA: Migraine with aura
MOA: Migraine without aura
MSQ: Migraine Screening Questionnaire
PIH: Post ictal headache
TTH: Tension type headache
