# Supplementary material for: The epidemiology of headaches among patients with epilepsy: a systematic review and meta-analysis
Source: J Headache Pain. 2020 Jan 10;21(1):3. doi: 10.1186/s10194-020-1074-0 (PMC6954535; doi:10.1186/s10194-020-1074-0)
Supplement: Supplementary file 1 — Additional file 1. The quality of studies included in systematic review and meta-analysis. [file 10194_2020_1074_MOESM1_ESM.docx]

**Supplementary file 1:** The quality of studies included in systematic review and meta-analysis

| Study name | NOS score | Quality level |
| --- | --- | --- |
| Erin K, 2017 (34) | 7 | Moderate |
| Singla et al., 2019 (35) | 8 | High |
| Sayena J et al., 2015 (36) | 8 | High |
| Ashjazadeh N, 2015 (37) | 9 | High |
| Fattahzadeh AG et al., 2017 (38) | 8 | High |
| Ito M et al., 2004 (26) | 8 | High |
| Inn-Chi L., 2018 (39) | 8 | High |
| Mainieri et al., 2015 (40) | 9 | High |
| Zhang et al., 2018 (37) | 5 | Low |
| Gökhan Ö et al., 2010 (43) | 8 | Moderate |
| Mameniškiene R et al., 2016 (44) | 9 | High |
| Wang et al., 2014 (25) | 9 | High |
| Slavica V. et al., 2012 (45) | 8 | High |
| Shamim R..,2018 (46) | 9 | High |
| Kwan P et al., 2008 (38) | 8 | High |
| Yosria AHA. et al., 2017 (43) | 7 | High |
| Sayed MA *et al, 2019 (44)* | 8 | High |
